# 4th year medical student elective in multidisciplinary thoracic oncology

**DOI:** 10.1186/1470-7330-15-S1-P23

**Published:** 2015-10-02

**Authors:** LE Quint, RM Reddy

**Affiliations:** 1 University of Michigan, 500 S State St, Ann Arbor, MI, USA

## Learning objective

The objectives of this poster are to describe the methodology and curriculum content employed by a senior medical student elective entitled “Multidisciplinary Thoracic Oncology.” This elective provides students with experience in a focused area of oncologic practice and is valuable to those pursuing careers in a variety of related fields.

## Content description

This month-long elective employs a flipped classroom approach with didactic teaching materials that are delivered on an interactive online platform. Materials include recorded videolectures with pre-lecture, post-lecture and embedded multiple choice questions (MCQs); case studies with a sequential, question and answer learning approach and embedded MCQs; a pre-course quiz and a post-course exam. These didactic materials are viewed by students at their own time and pace, typically during off hours. During daytime hours, the students rotate through relevant clinics, seeing patients and reviewing their cases with attending faculty, and occasionally assisting in surgical/interventional procedures. The areas covered include Medical Oncology, Pathology, Pulmonology, Radiation Oncology, Radiology and Thoracic Surgery, focusing on patients with lung, esophageal and other thoracic neoplasms. Each student presents one or more patients at the weekly Thoracic Tumour Board meeting. The capstone project is a written case study or an oral presentation based on a relevant topic. Comments from students who took this elective during the past year indicated enjoyment of: “full engagement in the learning process and the patient care team” and “obtaining a well-rounded, multidisciplinary understanding of the presentation and treatment of these patients.”

